# Exploring a Proposed WHO Method to Determine Thresholds for Seasonal Influenza Surveillance

**DOI:** 10.1371/journal.pone.0077244

**Published:** 2013-10-11

**Authors:** Ee Laine Tay, Kristina Grant, Martyn Kirk, Anthony Mounts, Heath Kelly

**Affiliations:** 1 Victoria Infectious Diseases Reference Laboratory, Melbourne, Australia; 2 National Centre for Epidemiology and Population Health, The Australian National University, Canberra, Australia; 3 Global Influenza Programme, World Health Organization, Geneva, Switzerland; National University of Singapore, Singapore

## Abstract

**Introduction:**

Health authorities find thresholds useful to gauge the start and severity of influenza seasons. We explored a method for deriving thresholds proposed in an influenza surveillance manual published by the World Health Organization (WHO).

**Methods:**

For 2002-2011, we analysed two routine influenza-like-illness (ILI) datasets, general practice sentinel surveillance and a locum medical service sentinel surveillance, plus laboratory data and hospital admissions for influenza. For each sentinel dataset, we created two composite variables from the product of weekly ILI data and the relevant laboratory data, indicating the proportion of tested specimens that were positive. For all datasets, including the composite datasets, we aligned data on the median week of peak influenza or ILI activity and assigned three threshold levels: seasonal threshold, determined by inspection; and two intensity thresholds termed average and alert thresholds, determined by calculations of means, medians, confidence intervals (CI) and percentiles. From the thresholds, we compared the seasonal onset, end and intensity across all datasets from 2002-2011. Correlation between datasets was assessed using the mean correlation coefficient.

**Results:**

The median week of peak activity was week 34 for all datasets, except hospital data (week 35). Means and medians were comparable and the 90% upper CIs were similar to the 95^th^ percentiles. Comparison of thresholds revealed variations in defining the start of a season but good agreement in describing the end and intensity of influenza seasons, except in hospital admissions data after the pandemic year of 2009. The composite variables improved the agreements between the ILI and other datasets. Datasets were well correlated, with mean correlation coefficients of >0.75 for a range of combinations.

**Conclusions:**

Thresholds for influenza surveillance are easily derived from historical surveillance and laboratory data using the approach proposed by WHO. Use of composite variables is helpful for describing influenza season characteristics.

## Introduction

Influenza infection remains a significant public health problem, resulting in considerable global morbidity and mortality [1-3]. In temperate regions of Australia, seasonal influenza outbreaks usually occur between late autumn and early spring and are associated with an increase in disease burden and utilisation of health service [3,4]. Due to differences in circulating viruses, population immunity and environmental factors, the onset, duration and severity of a season may differ from year to year [2,5]. Ongoing monitoring of influenza is therefore needed to determine the onset and severity of seasons and to monitor changes in disease trends. Surveillance which involves laboratory testing can add to data on virus characteristics.

Influenza thresholds have been developed to indicate a level of disease activity that would signal the start or end of a season or provide an alert to an unusually severe or atypical season. The onset of a season may stimulate diagnosis, enhance case detection, promote awareness of the need for patient cohorting or isolation in hospitals, remind people about vaccination and encourage early prescription of anti-viral medication, especially in vulnerable populations [6,7]. In the setting of a particularly severe season or pandemic, thresholds may inform the appropriate allocation of resources [8]. Many influenza surveillance systems around the world have incorporated the use of thresholds. These include Australia, New Zealand, Europe and United States (US) [9-12].

Methods using a variety of surveillance systems have been developed to establish thresholds for influenza activity. The methods vary in their complexity and can use either short-term or longer historical data to create time-varying or fixed thresholds. There is currently no gold standard or consensus for calculating thresholds. The simplest method uses visual inspection of historical data to create a fixed threshold used throughout the year [13]. Other methods include regression models [14-17], time series methods [15], calculation of means and medians [18-20] and adaptation of industrial control processes such as Shewhart charts [21], Cumulative Sum (CuSum) [15,18,22] and the Exponentially Weighted Moving Average [23]. The US Centre for Disease Control and Prevention (CDC) calculates the baseline for their influenza-like-illness (ILI) surveillance by adding two standard deviations to the mean percentage of ILI visits during non-influenza weeks for the previous three seasons, with non-influenza weeks defined as periods with less than 2% of the year’s total positive specimens for influenza for ≥ 2 consecutive weeks [24]. In Victoria, the General Practice Sentinel Surveillance (GPSS) for ILI has historically relied on thresholds determined by inspection [13].

In 2012 a novel but simple method for defining thresholds was proposed by the World Health Organization (WHO) as part of the development of global standards for influenza surveillance [7]. The proposed method aligns several years of historical data on the median week of peak activity and assigns thresholds based on means and standard deviations of aligned data. To our knowledge, this method has not yet been field tested.

The aim of this study was to explore the feasibility of the WHO method for the calculation of influenza thresholds using a range of existing surveillance and laboratory data sources in one surveillance system. We used these data sources to compare the onset, duration and intensity of influenza seasons.

## Methods

### Study setting

Victoria is a state with a population of over 5 million people located in the south-eastern part of Australia. It has a temperate climate with annual seasons of influenza occurring occur between late autumn (May) and early spring (October). It has a well-established influenza surveillance system that monitors influenza activity using syndromic surveillance of ILI presentations to sentinel general practitioners (GP) and a medical locum service; laboratory-confirmed influenza; hospital admissions for influenza; and more recently Google Flu trends and the influenza complications alert network (FluCAN), which monitors hospitalised patients from sentinel Australian hospitals, including four Victorian hospitals [9].

### Data sources

Four independent surveillance data sources were used: (i) the Victorian GPSS, (ii) sentinel data from the Melbourne Medical Deputising Service (MMDS), (iii) routine laboratory-confirmed influenza (LAB data) from the Victorian Infectious Diseases Reference Laboratory (VIDRL) and the (iv) Victoria Admitted Episode Dataset (VAED) for hospital admissions.

The GPSS is an annual surveillance system for ILI and laboratory confirmed influenza that was established in 1993, with laboratory support added in 1998 [25]. Surveillance extends from week 18 to 44 each year during the influenza season. The number of participating general practitioners (GP) has varied from 40 to 100 since the scheme’s establishment. The ILI definition used is based on the nationally agreed case definition of cough, fever (measured or reported) and fatigue [13]. Approximately 48% of ILI patients seen by sentinel GPs were swabbed and of these, influenza virus was detected from an average of 34% (18%-47%) of the swabbed ILI patients tested from 2003 to 2011 [9,26].

An alternative source of community sentinel ILI surveillance is the MMDS, an out-of-hours medical locum service that covers an approximate 45km radius from central Melbourne. GPs from the deputising service consult with patients in their own home or aged care facility. The diagnosis made by the attending doctor is recorded electronically and de-identified summary data are available on a password protected website within 24 hours. ILI data are extracted weekly based on a previously developed search algorithm [27]. There is no laboratory support for the MMDS surveillance which has nonetheless been shown to provide equivalent information to surveillance data from sentinel general practitioners [28].

LAB data from VIDRL consist of laboratory detections of influenza viruses from all routine respiratory samples sent to VIDRL, tested using an in-house respiratory multiplex reverse transcriptase polymerase chain reaction (RT-PCR) test that identifies influenza viruses, adenovirus, picornavirus, respiratory syncytial virus, parainfluenza virus, coronavirus and human metapneumovirus [29]. Many of the samples are referred from major adult teaching hospitals in Victoria [30].

The VAED is a hospitalisation dataset on all patients admitted to public and private acute care hospitals in the state of Victoria. The clinical information coded for each episode of care is based on the International Classification of Diseases and Related Health Problems, Tenth Revision, Australian Modification (ICD-10-AM). We extracted records containing influenza codes J09-11 in primary or secondary diagnostic fields.

In addition, a further two composite variables were created from the product of ILI and LAB data. These were the

GPSS composite = proportion of ILI cases in GPSS x proportion of laboratory samples tests positive for influenza in GPSS

MMDS composite = proportion of ILI cases in MMDS x proportion of routine laboratory tests positive for influenza in LAB data.

While the GPSS proportion and laboratory testing are part of the same system, the MMDS is independent of routine clinical tests referred to VIDRL. However, both MMDS and routine VIDRL clinical testing focus on older age groups and were matched based on age profiles [30,31].

The metrics used for threshold calculations were the weekly GPSS ILI proportion per 1000; the weekly MMDS ILI per 1000; the proportion of laboratory test positive for influenza using the total number of tests for influenza as the denominator (LAB test positive influenza); the weekly proportion of influenza admissions in the population using the mid-year estimated resident population in Victoria as the denominator and expressed per 100,000 population [32]; and the product of weekly ILI and LAB data per 1000. We preferred the use of test positive influenza to count data to compensate for changes in testing behaviour over time [33].

### Data extraction

Depending on availability, data were extracted from 2002 to 2011 for GPSS and MMDS, 2003 to 2011 for LAB data and 2005 to 2011 for VAED. For MMDS, LAB data and VAED, data were complete for all 52 weeks but only weeks 18 to 44 were available for GPSS. For VAED, only aggregated data containing admissions of more than five counts were provided to protect privacy and confidentiality of individuals.

### Methods for determining thresholds

We defined the three levels of influenza threshold based on the terminology used in the WHO manual and the existing Victorian surveillance thresholds that had been adapted from the United Kingdom: seasonal, average and alert [13,34]. Seasonal threshold defines the start and end of an influenza season. The two intensity thresholds, termed average and alert thresholds, describe relative seasonal intensity.

The WHO manual did not prescribe a specific method for determining the seasonal threshold, that is, the start of the season. We used the method of inspection of the complete data for the six datasets to determine the seasonal threshold. For each dataset this was done independently by four of the co-authors (ET, KG, AM, HK) and differences were resolved by discussion. For the four datasets that provided data for the whole year (MMDS, Lab data, VAED and MMDS composite), we calculated the 95% confidence interval (CI) of the metrics used for each dataset for the period defined as out-of-season, that is, the values below the seasonal threshold, that had been determined by inspection. We also explored the seasonal threshold using the 95th percentile of out-of-season data without assuming data were normally distributed. We then compared the seasonal threshold set by inspection with the 95% CI and 95th percentiles of the average out-of-season values.

**Figure 1:**
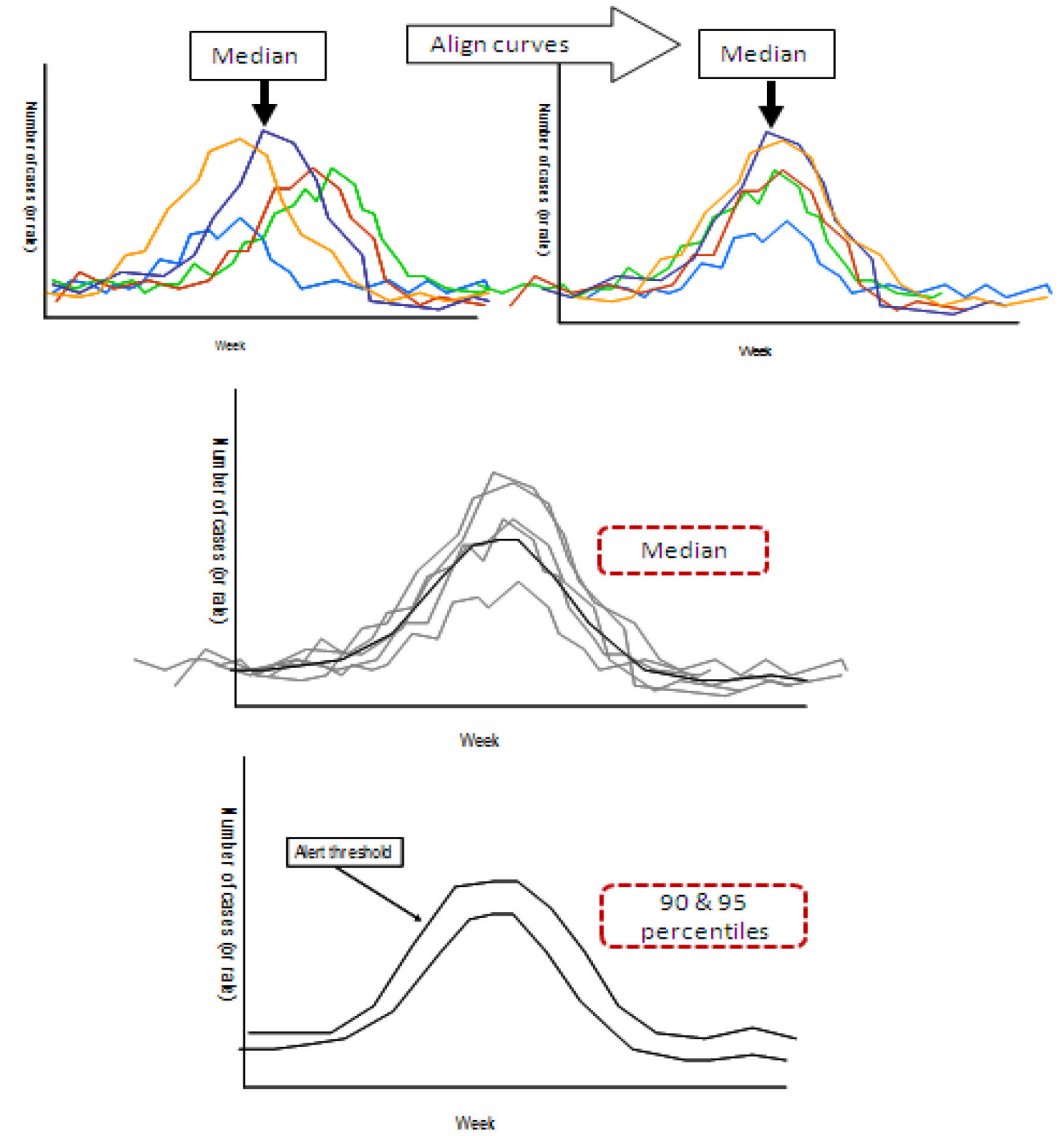
WHO method for determining thresholds. Figure adapted from the WHO Global Surveillance Standards for Influenza [7].

Average and alert thresholds were calculated for each dataset using a variation of the WHO protocol [7] (Figure 1). We first determined the median week of peak occurrence using historical data, excluding the pandemic year of 2009 which was atypical from both surveillance and testing perspectives [33]. We then aligned the transmission peaks around the median week of peak occurrence (Figure 1A) and calculated the weekly mean and standard deviations for each week centred on the median week of peak occurrence (Figure 1B and C).

The WHO protocol suggests the use of the Normal distribution to assign thresholds based on the mean and standard deviation of the aligned data for weekly counts. However, we believed data were unlikely to be Normally distributed for all years and tested this by inspection and formally using the Shapiro-Wilks test for Normality for GPSS, MMDS, LAB data and the VAED for each year during season [35].

In addition to the mean and standard deviations, we explored the thresholds using the median and 90th and 95th percentiles. The average threshold was determined by a comparison of the peak weekly mean and median, while the alert threshold was determined by a comparison of the peak weekly upper 90% and 95% CI upper limits with the 90th and 95th percentiles. We also performed log transformation of all datasets and calculated the corresponding geometric mean and 90/95 CI upper limit.

### Comparison of thresholds

Once the seasonal thresholds were assigned, we determined the start and end of each season independently for all datasets, each defined as the two consecutive weeks where the seasonal threshold was crossed. We used the average and alert thresholds to categorise the influenza seasons, based on the threshold range of peak seasonal activity, specifically seasonal-average, average-alert or alert. Comparisons were made for the onset, duration and intensity of a season across all datasets from 2005-2011 based on data availability. We also compared how all seasons compared to the average season created using aligned historical data described above.

Finally, to determine how correlated the six datasets were, we calculated the correlation coefficient for each year from 2005 to 2011 for a combination of datasets, from which a mean correlation coefficient and its corresponding 95% confidence limits was derived.

Data were analysed using Microsoft® Office Excel 2003 and Stata version 10.0 (Stata Corp., College Station, TX, USA).

### Ethics Statement

The study was approved as a quality assurance project by the Melbourne Health Office of Research. GPSS and MMDS data in this study were collected, used and reported under the legislative authorization of the Victorian Public Health and Wellbeing Act 2008 and Public Health and Wellbeing Regulations 2009.

## Results

### Determination of thresholds

During the study period, the highest number of ILI or influenza cases annually ranged from 56 to 208 per week for the GPSS; 33 to 164 per week for the MMDS; 24 to 135 per week for test positive influenza; and 28 to 204 per week for influenza admissions.

Testing for normality of the weekly count data for each year suggested no seasonal surveillance data had a classical Normal distribution graphically (data not shown). Data were consistent with a Normal distribution by formal testing for 5/10 seasons in the GPSS, for 5/10 seasons in the MMDS, for 2/9 seasons in LAB data and for 2/6 seasons in the VAED. When data were log-transformed, the number of seasons with Normal distribution increased (8/10, 8/10, 5/9 and 3/6 respectively).

**Figure 2:**
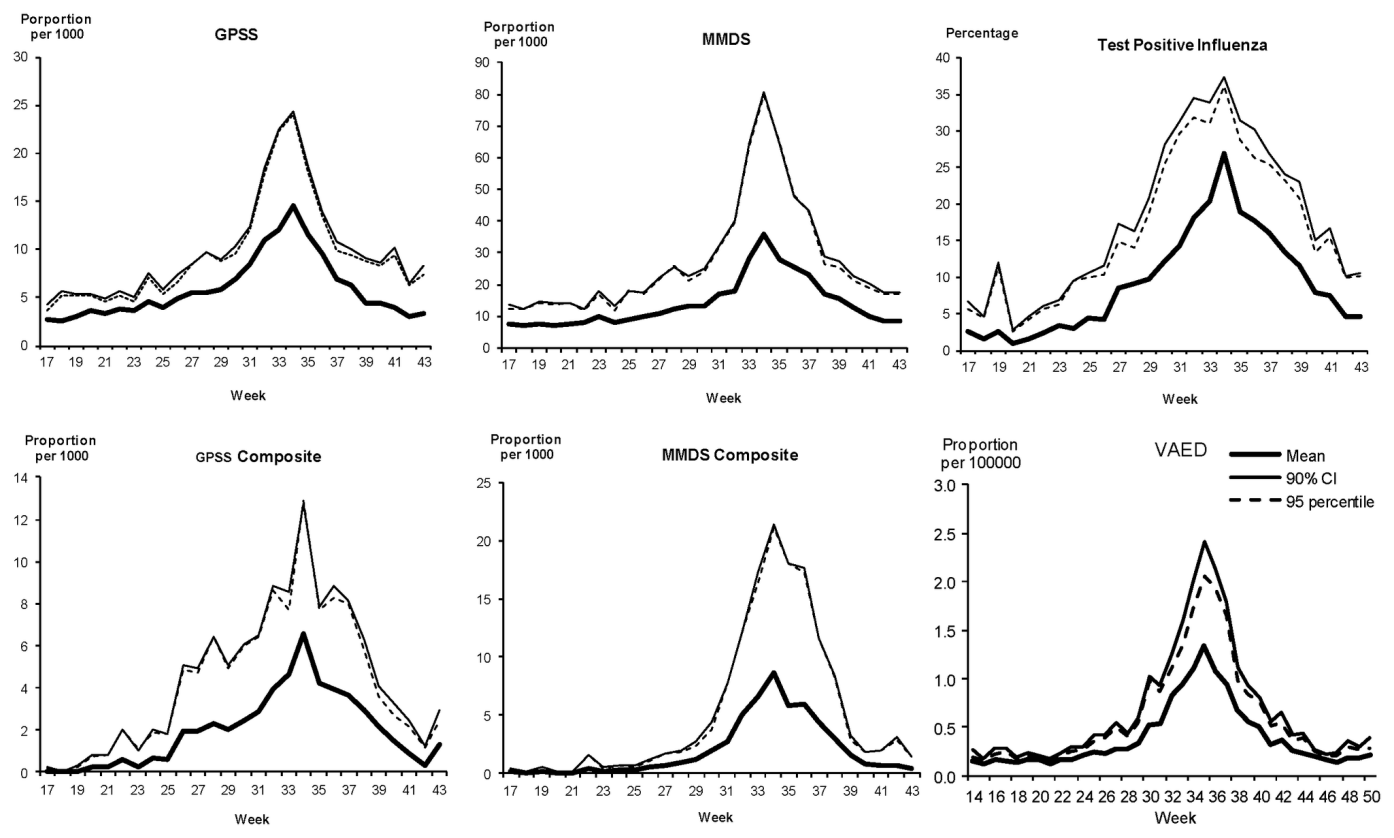
Mean, upper 90% Confidence Interval and 95th percentiles for six surveillance datasets.

The threshold parameters from the adapted WHO method are summarised in Table 1. The median week of peak occurrence for all datasets was week 34 except for the MMDS composite and VAED. The values assigned for seasonal thresholds by inspection were similar to the 95% CI upper limit and 95^th^ percentile of the average out-of-season values for all datasets. For the average threshold, we found the peak mean values for the GPSS, test positive influenza and VAED were similar to the median but the peak mean was higher for the MMDS and MMDS composite due to the high call out proportion in 2003. We therefore use the peak mean to define the average threshold (Table 1). To set the alert thresholds, the peak 90% CI upper limit was used as we found the parameter to be similar to the peak 95^th^ percentile across all six datasets (Table 1 and Figure 2). The geometric means and 90% CI upper limits from log-transformed data also produced similar parameters (data not shown).

**Table 1:** Comparison of parametric and non-parametric parameters and thresholds for six surveillance datasets.

**Dataset**	GPSS^*^	MMDS^*^	**Test Positive Influenza^†^**	GPSS composite^*^	MMDS Composite^*^	**VAED^‡^**
***Seasonal Threshold***						
Seasonal threshold (inspection)	4.0	10.0	5.0	1.5	1.0	0.3
95% CI (out of season)	4.3	10.2	4.5	1.4	0.7	0.3
95th percentile (out of season)	3.9	9.7	4.4	1.4	0.7	0.3
***Average and Alert Thresholds***						
Median week of peak occurrence	34	34	34	34	34.5	35
*Range (weeks)*	24-40	26-37	27-44	30-39	25-41	29-38
Peak Mean	14.5	35.7	26.9	6.6	8.6	1.3
*Range*	8.9-25.4	13.8-104.2	20.0-37.1	3.3-14.6	2.3-24.8	0.6-2.1
Standard deviation	6.0	27.4	6.4	3.8	7.8	0.7
*90% CI upper limit*	24.3	80.8	37.4	12.8	21.4	2.4
*95% CI upper limit*	26.2	89.4	39.4	13.9	23.8	2.6
Peak Median	13.4	22.9	24.6	5.5	4.8	1.4
*90th percentile*	24.1	55.0	35.0	11.2	17.5	2.0
*95th percentile*	22.6	79.6	36.1	12.9	21.1	2.1
***Threshold levels^§^ Categories of influenza season***	**Threshold values**					
< Seasonal	<4.0	<10.0	<5.0	<1.5	<1.0	<0.3
Seasonal - Average	4.0-15.0	10.0-35.0	5.0-27.0	1.5-6.5	1.0-8.5	0.3-1.3
Average - Alert	>15.0 - 24.0	>35.0 - 80.0	>27.0 - 37.0	>6.5 - 12.0	>8.5 - 20.0	>1.3 - 2.4
> Alert	>24.0	>80.0	>37.0	>12.0	>20.0	>2.4

* Expressed as proportion per 1000; † Expressed as percentage; ‡ Expressed as proportion per 100000; § Seasonal threshold set using visual inspection, average threshold using the mean and alert threshold using the 95% CI upper limit.

### Comparison of thresholds

**Figure 3:**
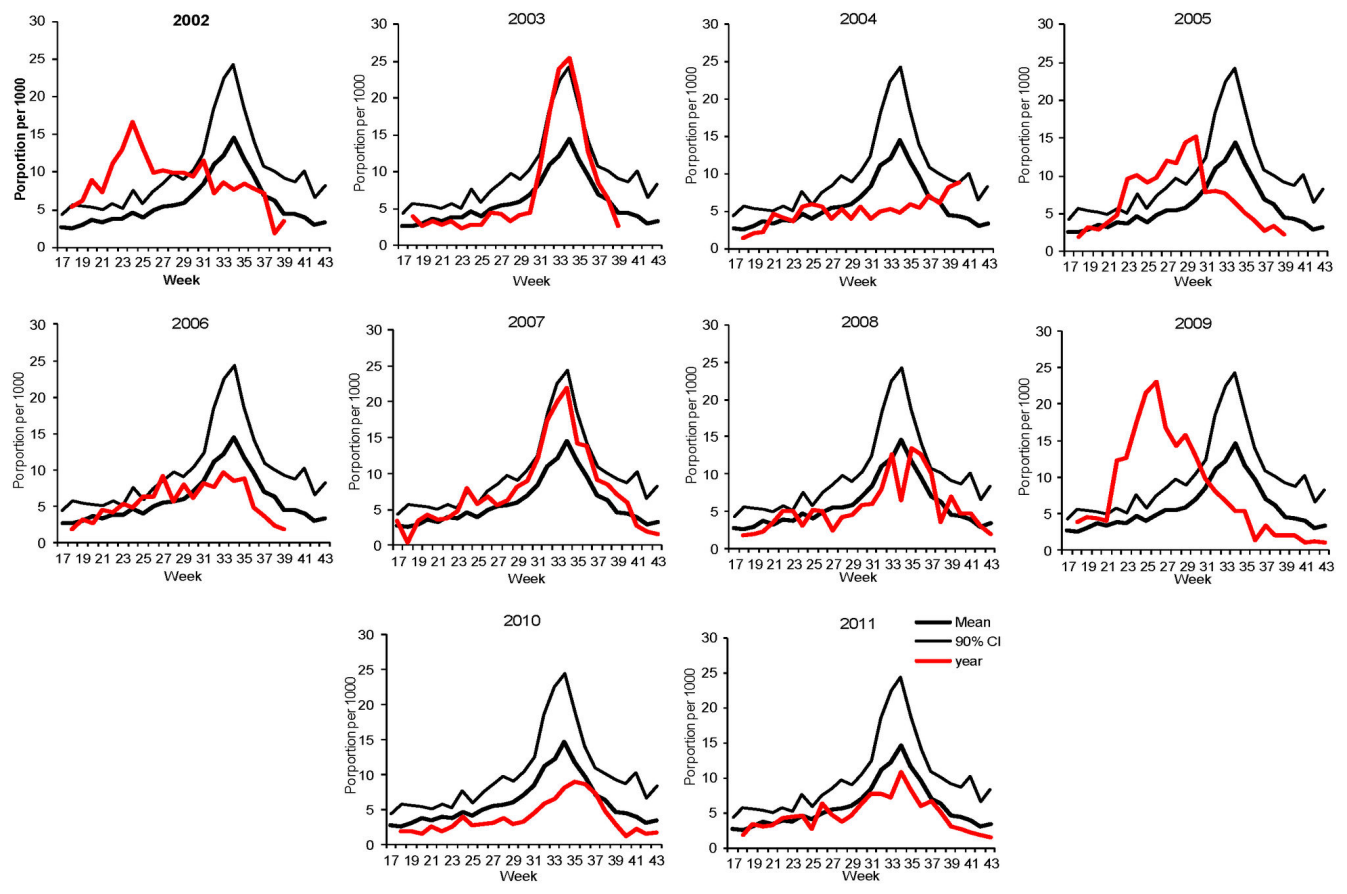
Annual data (red) plotted against the ten year average season (black) for the GPSS, 2002-2011.

Using the GPSS dataset as an example, Figure 3 compares how an annual season compares against the average season calculated using ten years of historical data.

#### Onset, end and duration of influenza season across all datasets

For the seven years where data were available for all datasets, GPSS assigned seasons tended to start much earlier for most years compared to other datasets. The use of the GPSS composite suggested a later start to the season. Season onset according to the VAED generally lagged behind other datasets for most years and was variable for the MMDS (Table 2).

**Table 2:** Onset, duration and intensity of influenza season, by year and surveillance dataset, Victoria, 2005 to 2011.

	**VAED Onset / End (duration)**	**GPSS Onset / End (duration)**	**MMDS Onset / End (duration)**	**Test Positive Influenza Onset / End (duration)**	**GPSS Composite Onset / End (duration)**	**MMDS Composite Onset / End (duration)**
***Onset and duration of seasons***						
**2005**	25/36 (12)	22/36 (15)	21/40 (20)	20/36 (17)	22/35 (14)	21/36 (16)
**2006**	25/36 (12)	21/36 (16)	20/35 (16)	21/38 (18)	25/36 (12)	24/35 (12)
**2007**	29/41 (13)	23/40 (18)	29/39 (11)	26/45 (20)	26/>43 (-^†^)	26/39 (14)
**2008**	32/42 (11)	22/41 (20)	35/40 (6)	28/44 (17)	31/41 (11)	33/40 (8)
**2009**	22/39 (18)	19/35 (17)	22/32 (11)	23/33 (11)	22/33 (12)	23/31 (9)
**2010**	31/46 (16)	31/38 (8)	33/38 (6)	34/41 (8)	31/38 (8)	34/38 (5)
**2011**	27/45 (19)	22/38 (17)	31/38 (8)	28/41 (14)	30/39 (10)	32/38 (7)
***Intensity of seasons***						
**2005**	A	A	B	B	B	B
**2006**	A	A	A	A	A	A
**2007**	B	B	B	B	B	B
**2008**	A	A	A	A	A	A
**2009**	C	B	B	-*	B	A
**2010**	B	A	A	A	A	A
**2011**	B	A	A	A	A	A

* Not determined due to the centralised influenza testing that occurred at VIDRL during the pandemic year; † Inadequate data to define the end of a season; A (Seasonal – Average), B (Average – Alert), C (Alert)

For most pre-pandemic years, there was generally good agreement for defining the end of a season across datasets. However, a divergent trend, not reflected in other datasets, was noted in the VAED from 2009 onwards (Table 2). The VAED assigned seasons ended much later, resulting in a longer assigned seasonal duration.

#### Category of influenza season

**Figure 4:**
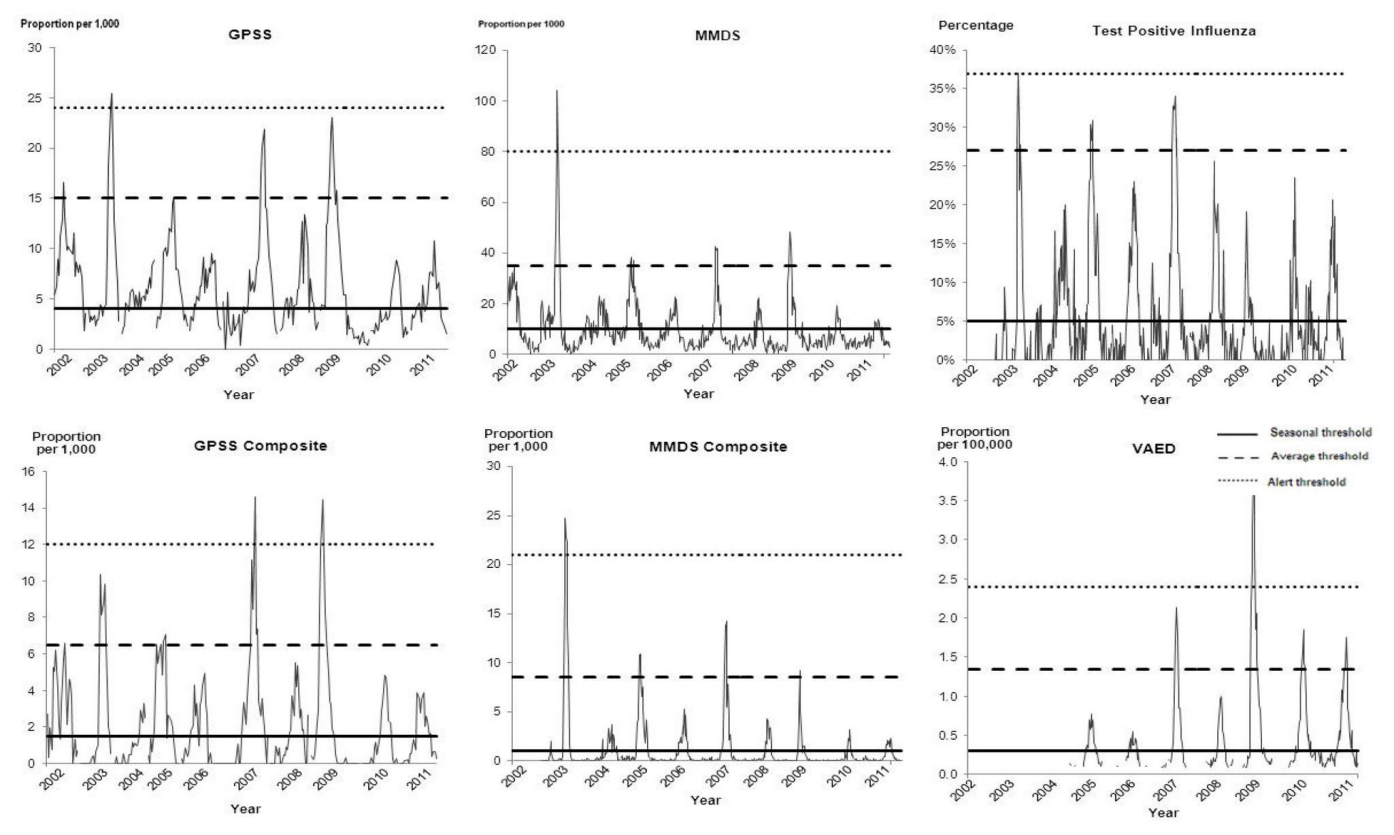
Categories of influenza season in Victoria for six surveillance datasets, 2002-2011.

There was agreement in describing the intensity of influenza seasons in 3/7 years prior to 2009 (Table 2 and Figure 4). During the pandemic year, the season intensity varied according to different data sources and from 2009 onwards, peak seasonal influenza activity was between the seasonal and average thresholds (or seasonal-average) for all datasets except the VAED.

### Correlation between datasets

Datasets were found to be well correlated, with mean correlation coefficients of >0.75 for a range of combinations (Table 3). Correlations between the VAED and other datasets improved once the VAED was aligned with other datasets to correspond to the one week lag in median week of peak occurrence**.**

**Table 3:** Correlation Coefficient between different datasets, 2005-2011.

**Dataset**		**95%CI**	VAED aligned^*^	
	**Mean**		**Mean**	**95%CI**
	**Correlation Coefficient**		**Correlation Coefficient**	
**VAED and GPSS**	0.77	0.62-0.92	0.82	0.69-0.95
**VAED and MMDS**	0.76	0.64-0.88	0.83	0.74-0.92
**VAED and Lab data**	0.79	0.67-0.92	0.79	0.66-0.92
**VAED and GPSS Composite**	0.77	0.61-0.92	0.78	0.63-0.94
**VAED and MMDS Composite**	0.80	0.64-0.95	0.82	0.70-0.94
**GPSS and MMDS**	0.78	0.67-0.90	-	-
**GPSS and MMDS Composite**	0.79	0.72-0.87	-	-
**GPSS and Lab data**	0.78	0.69-0.86	-	-

* **VAED aligned with other datasets to correspond to the one week lag in median week of peak occurrence.**

## Discussion

Thresholds for influenza surveillance were easily derived using a simple method proposed by the WHO. The method was adapted to a non-parametric approach that produced similar findings to the suggested protocol based on the Normal distribution. Log transformation of the data produced comparable findings to both approaches. Comparison of thresholds derived from different datasets revealed variations in defining the start of a season but relatively good agreement in describing the end and intensity of influenza seasons, except in the hospital data after the pandemic year.

As the WHO protocol does not prescribe a method for defining the seasonal threshold, we used the simplest method of visual inspection but showed that the levels were consistent with variation in out-of-season virus circulation. Numerous other approaches exist, based on more complex statistical techniques but many of these approaches usually require a pre-determined threshold to be nominated [15,21,23], again often by inspection.

In the setting of the average and alert thresholds for our datasets, we used the peak mean values to set the intensity thresholds as per the WHO protocol after observing the data agreement between the peak weekly means and medians and 90% CI upper limit and 95 percentiles. However, the median and 90 or 95 percentiles may be a more appropriate option when data are not Normally distributed and no transformation has been performed. In practice, another point of consideration for the setting of the alert threshold may be a level of influenza activity that corresponds to an increased demand on the health care system [7]. This would be dependent on local health care systems. We also incorporated a two week consecutive rule into the definition of the onset and intensity of a season to reduce the number of false positive signals.

Based on the seasonal thresholds, we found inconsistencies in defining the start and end of a season across the datasets. Given the variations in timeliness of influenza reporting [31], we would expect the onset of ILI surveillance to precede laboratory confirmed influenza and hospital admissions, and that both the ILI surveillance systems might coincide with one another. By incorporating a laboratory component to the ILI measure, the use of the composite variable appeared to improve the specificity and agreement between VAED and ILI surveillance data. The finding is consistent with emerging literature that these composite variables may be a better proxy indicator of influenza incidence than either ILI or LAB data alone [36,37]. The use of composite variables in surveillance warrants further investigations.

In comparing the intensities of an influenza season, there was good agreement across all datasets, except for the VAED after 2009. The number of hospital admissions coded for influenza has increased both in and out of the influenza season, with the duration of the season prolonged due to the late end signal. These changes were not reflected in the ILI or LAB datasets. While there may be a number of possible explanations, such as changes in testing behaviours or disease coding, a recent study investigating the increase in out-of-season influenza in Australia suggests a genuine increase in influenza activity, combined with increased testing that occurred following the pandemic [38]. This may reflect an increased awareness of influenza in hospitalised patients among health professionals after the pandemic. Additionally, surveillance data at VIDRL indicate that approximately 40% of patients with an influenza-like illness were swabbed prior to 2009 [26] but this rose to 70% during the 2009 pandemic [39] and has since remained at about this proportion [9].

The measurement of the intensity a season was based on the peak of influenza activity and whether or not the thresholds were exceeded for two weeks. This reflects only a single dimension of measurement and does not take into account how long the influenza activity remained within a particular category or the rate of increase in the number of cases. For example, a short-term acute rise in influenza cases that marginally exceed the alert threshold does not correspond to a gradual or persistent elevated level of activity that may represent a higher disease burden*.*

Finally, when we compared the current parameters to the previous threshold based on seven years of historical ILI data from 1994-2000 in Victoria, the baseline threshold for the GPSS was lower at 2 per 1000 cases compared with the revised threshold of 4 and the alert threshold was higher at 35 per 1000 cases compared with the revised threshold of 24 [13]. These differences indicate the need of regular review of surveillance-derived thresholds.

In conclusion, this study has shown that the proposed WHO threshold protocol is simple to implement and could be easily adapted for any influenza surveillance system with adequate historical data. However, the study was based in a region with a temperate climate, and its application in the tropics would require further work. Further exploration of the proposed WHO method in another temperate region would be of interest. While thresholds are useful as a warning system, they should always be interpreted with other available information.
